# 非小细胞肺癌患者中Survivin抗体的临床意义及诊断价值

**DOI:** 10.3779/j.issn.1009-3419.2010.07.09

**Published:** 2010-07-20

**Authors:** 丽 马, 文涛 岳, 丽娜 张, 玥 王, 春彦 张, 学惠 杨

**Affiliations:** 101149 北京，北京市结核病胸部肿瘤研究所，北京市肿瘤分子生物学实验室肺癌分室（细胞生物学研究室） Department of Cellular and Molecular Biology, Beijing TB and Thoracic Tumor Research Institute, Beijing 101149, China

**Keywords:** 原核表达, Survivin自身抗体, 肺肿瘤, 间接ELISA, 联合检测, Prokaryotic expression, Anti-survivin antibody, Lung neoplasms, Indirect ELISA, Combined detection

## Abstract

**背景与目的:**

在世界范围内，恶性肿瘤已经成为威胁人类健康的主要原因，且其发病率和死亡率居高不下。随着人们对肿瘤标志物研究的不断深入，肿瘤相关的自身抗体成为了研究热点。肺癌患者血清中Survivin自身抗体的临床意义目前还存在争议。本研究旨在探讨Survivin自身抗体在非小细胞肺癌患者血清中可能的临床应用价值。

**方法:**

采用RT-PCR方法获得Survivin cDNA，构建原核表达载体pET30a(+)/Survivin，亲和层析方法纯化蛋白，SDS-PAGE电泳及Western blot鉴定，建立基于Survivin融合蛋白的间接ELISA方法，对89例健康志愿者、215例非小细胞肺癌患者以及20例肺部良性疾病患者的血清样本进行检测。

**结果:**

重组Survivin融合蛋白在BL21(DE3)中以包涵体形式高效表达，间接ELISA方法检测Survivin自身抗体在非小细胞肺癌患者血清中的阳性率为19.5%，特异性为88.9%，Survivin自身抗体与非小细胞肺癌患者的肿瘤大小、远处转移间存在相关性（*P* < 0.05），Survivin自身抗体与CEA在非小细胞肺癌患者中联合检测的阳性率明显高于CEA与NSE、SCC、CYFRA、ProGRP联合检测的阳性率，这大大提高了非小细胞肺癌检测的敏感性。

**结论:**

本研究成功构建原核表达载体pET30a(+)/Survivin，并建立检测Survivin自身抗体的间接ELISA方法，Survivin自身抗体与肺癌肿瘤大小、远处转移间的相关性及在非小细胞肺癌患者联合检测中的重要作用，为Survivin自身抗体在肺癌中的临床应用提供了线索和依据。

## Abstract (en)

**Background and objective:**

Lung cancer is one of the most common cancers worldwide and causes more deaths per year than other cancers. Autoantibody was one hotspot in tumor marker research. But the clinical value of Survivin autoantibody is not clear in lung cancer patients at present. The aim of this study is to dertermine whether Survivin autoantibody could be used as a diagnostic factor of lung cancer and what the clinical value of Survivin autoantibody was in non-small cell lung cancer (NSCLC).

**Methods:**

Survivin cDNA sequence was obtained by RT-PCR and inserted into prokaryotic expression vector pET30a(+). Fusion protein of Survivin was expressed in *E.coil* BL21(DE3). SDS-PAGE and Western blot were used to confirm the fusion protein. The Survivin protein was purified by Ni-NTA agarose affinity chromatography. At last, indirect ELISA was used to detect Survivin autoantibody level in the serums of 215 samples from NSCLC patients and serums from 20 samples of nonmalignant lung disease patients as well as 89 samples of healthy persons were also detected as control.

**Results:**

The recombinant vector pET30a(+)/Survivin was contructed and the Survivin protein is successfully expressed in *E.coil* BL21(DE3) as inclusion body. Indirect EILSA was done to detect Survivin autoantibody in these serums using purified Survivin protein. It was shown that the positive rate of Survivin autoantibody was 19.5%, with a specificity of 88.9% in NSCLC patients. The expression of Survivin autoantibody has correlation with the volume of tumor and the metastasis of tumor in NSCLC patients (*P* < 0.05). In our research, positive detection rate of NSCLC was much improved by detecting Survivin autoantibody combined with CEA compared to other tumor markers combined with CEA.

**Conclusion:**

In this study, purified fusion protein of Survivin was successfully obtained. Indirect ELISA for detecting Survivin autoantibody in serum of NSCLC patients using purified Survivin protein was established. Our results indicated that Survivin autoantibody as a latent value of tumor marker could be used for clinical diagnosis in NSCLC patients.

Survivin作为凋亡抑制蛋白（inhibitor of apoptosis proteins, IAP）家族的重要成员，在肿瘤的发生发展过程中发挥重要作用。Survivin几乎表达于所有的恶性肿瘤，但在人体正常终末分化的组织细胞中不表达或低表达。肿瘤自身抗体产生的机制虽然还不明确，但是同肿瘤相关抗原一样，其与肿瘤的发生发展存在密切的联系。据研究显示在多种癌症血清中可以检测到Survivin自身抗体的存在，如头颈癌、卵巢癌^[1]^以及消化系统肿瘤胃癌^[2]^、结肠直肠癌^[3]^、肝癌^[4]^等。在肺癌患者血清中同样可以检测到Survivin自身抗体^[3, 5]^，但Survivin自身抗体能否成为具有肺癌诊断价值的肿瘤标志物以及与肺癌病理参数是否存在相关性，目前尚不统一。本研究旨在以Survivin融合蛋白原核表达为基础，建立肺癌患者血清中Survivin自身抗体的间接ELISA方法，探讨Survivin自身抗体在215例非小细胞肺癌（non-small cell lung cancer, NSCLC）患者中可能存在的临床意义及诊断价值。

## 1 材料与方法

### 1.1 细胞系及菌株

肺癌细胞系A549、E.coil菌株BL21(DE3)以及pET30a(+)载体为北京市结核病胸部肿瘤研究所细胞生物学实验室冻存。

### 1.2 材料

pGEM-Teasy、T4 DNA连接酶、高保真酶PCR试剂盒购自NEB公司；限制性内切酶*Nde*Ⅰ和*Xho*Ⅰ购自宝拓科技发展有限公司；逆转录酶、Oligo(dT)15 Primer购自Promega公司；镍离子亲和层析柱购自威格拉斯生物技术（北京）有限公司；Survivn单克隆抗体购自优宁维公司；PCR引物合成及DNA测序由上海生工北京分公司生物技术有限公司完成。

### 1.3 研究对象

本研究使用的血清标本于2008年3月-2009年12月由北京市结核病胸部肿瘤研究所标本库收集并提供。89例健康志愿者，20例肺部良性疾病，包括10例肺结核、6例肺炎、2例支气管扩张症、2例错构瘤；215例NSCLC患者，男性147例，女性68例，平均年龄59.25岁，其中 < 60岁106例，≥60岁109例，年龄最大84岁，最小30岁。鳞癌83例，腺癌132例，临床分期：依据1997年国际抗癌联盟（UICC）TNM分期标准，其中Ⅰ期-Ⅱ期44例，Ⅲ期-Ⅳ期161例。全组血清收集标准：健康对照组均身体健康，体检无异常表现，临床各项生化指标正常；肺癌患者血清标本以及肺部良性疾病患者血清标本均采集自首次到北京市胸科医院就诊，未经任何化疗和治疗，且肿瘤类型均经组织病理学证实，标本分装后于-80 ℃冻存。

### 1.3 方法

#### 1.3.1 RT-PCR

取5×10^6^对数生长期肺腺癌细胞系A549细胞，Trizol一步法提取总RNA，使用Access RT-PCR System（Promega A1280）反转录试剂盒，按照说明书操作获得cDNA。

根据Survivin基因组序列用primer premier 5软件设计引物，在正、负链的5'端分别引入*Nde*Ⅰ和*Xho*Ⅰ酶切位点，正义链：5'-TTAACTCGCGATCCATGGCAGCCAGC-3'，反义链：5'-AATACATATGATGGGTGCCCCGACG-3'）。取cDNA模板2 μL、2 U/μL高保真DNA聚合酶1 μL、10 mmol/L dNTP 1 μL、10 mmol/L上、下游引物各1 μL、10×PCR buffer 5 μL，补水至50 μL。热启动95 ℃、5 min，循环参数为94 ℃、30 s，56 ℃、45 s，72 ℃、1 min，共30个循环，72 ℃延伸10 min，4 ℃终止反应。

#### 1.3.2 表达载体pET30a(+)/Survivin的构建和鉴定

PCR产物克隆入载体pGEM-Teasy中，转化感受态细胞E.coli DH5α，蓝白斑筛选，限制性内切酶*Nde*Ⅰ/*Xho*Ⅰ双酶切初步鉴定重组质粒，挑选阳性克隆菌株，扩增并提取pGEM-Teasy/Survivin质粒。*Nde*Ⅰ/*Xho*Ⅰ双酶切pGEM-Teasy/Survivin和pET30a(+)，65 ℃、20 min酶灭活，切胶回收酶切片段。pET30a(+) 1 μL、pGEM-Teasy/Survivin 3 μL、T4连接酶1 μL、2×T4连接buffer 5 μL，4 ℃连接过夜，构建表达载体pET30a(+)/Survivin，并转化感受态细胞*E.coli* DH5α，挑取克隆菌株，扩增提取质粒，PCR及双酶切鉴定的阳性克隆菌株送往上海生物技术有限公司北京分公司进行测序，将测序结果正确的pET30a(+)/Survivin转化入*E.coli* BL21(DE3)中，用含45 μg/mL卡那青霉素LB固体培养基37 ℃过夜培养。

#### 1.3.3 pET30a(+)/Survivin在*E.coli* BL21(DE3)中的诱导表达

挑取pET30a(+)/Survivin/*E.coli* BL21(DE3)克隆菌斑，37 ℃、250 rpm过夜振荡培养，按照1:100比例接种于LB-kan+液体培养液（含30 μg/mL卡那青霉素的LB），37 ℃、250 rpm培养至OD_600_约0.6-0.8时，以终浓度为0.1 mmol/L、0.2 mmol/L、0.3 mmol/L、0.4 mmol/L、0.8 mmol/L、1 mmol/L IPTG诱导菌液3 h，分别取1 mL菌液，12 000 rpm离心1 min收集菌体，取50 μL 2×loading buffer与菌体充分混匀、100 ℃水浴5 min，离心后取10 μL上清用于12% SDS-PAGE电泳分析，筛选最适IPTG诱导浓度，同时设空载体pET30a(+)以及未诱导pET30a(+)/Survivin作为阴性对照。

#### 1.3.4 Survivin融合蛋白的Western blot鉴定

取最适IPTG诱导菌液样本10 μL，经12% SDS-PAGE电泳分离后，电转移至硝酸纤维膜上，5%脱脂奶粉4 ℃封闭过夜，分别用1:500抗His抗体、1:1 000 Survivin单克隆抗体37 ℃孵育2 h，PBST洗涤3次×10 min，1:10 000羊抗小鼠抗体-HRP 37 ℃孵育1 h，PBST洗涤3次×10 min，ECL显色，Western blot分析。

#### 1.3.5 包涵体的制备及蛋白纯化

利用细胞分层分级技术，确定Survivin融合蛋白的表达形式。500 mL细菌培养液在最适IPTG浓度下诱导培养，收集菌体，冰浴超声破碎，7 000 rpm、4 ℃离心20 min，收集沉淀，溶解于含15 mL 6 mol/L盐酸呱、10 mmol/L Tris·Cl（pH7.0）、10 mmol/L DTT的变性液中，4 ℃过夜。次日7 000 rpm、4 ℃离心20 min，收集上清，0.45 μm滤膜过滤，4 ℃下以0.5 mL/min速度加入镍离子亲和层析柱中，而后分别用20 mmol/L、30 mmol/L、50 mmol/L、100 mmol/L、200 mmol/L咪唑溶液洗脱目的蛋白。收集各洗脱峰流出液，分别取10 μL样本100 ℃水浴5 min，经12% SDS-PAGE电泳确定并回收含有Survivin融合蛋白的洗脱液，4 ℃下PBS（pH7.4）透析3 h，冷冻干燥所得的蛋白干粉于-20 ℃保存。

#### 1.3.6 间接ELISA方法的建立和血清样本的检测

Survivin蛋白（纯度 > 95%）以15 μg/mL溶于0.05 mol/L碳酸盐缓冲液（pH9.6）中，100 μL/孔，4 ℃静置过夜；弃去板孔内液体，200 μL PBST洗板3次；100 μL 5%脱脂奶粉37 ℃封闭1 h；肿瘤患者血清1 μL/孔，37 ℃孵育2 h；200 μL PBST洗板3次；100 μL 1:10 000稀释的羊抗人IgG-HRP，37 ℃孵育1 h；200 μL PBST洗板4次；100 μL TMB显色液，37 ℃避光显色5 min；100 μL终止液终止显色反应，自动酶标仪上读取450 nm处OD值。PBS作为阴性对照孔，1:400 Survivin单克隆抗体作为阳性控制孔，10份健康人血清标本作为阴性控制孔，帮助评价不同孔板间的差异以减少板间误差，空白孔调零。

#### 1.3.7 数据处理

本实验中血清样品OD值均取复孔均值；采用SPSS 13.0软件进行数据整理和统计分析，χ^2^检验用于计数资料统计分析，以*P* < 0.05为差异具有统计学意义。

## 2 结果

### 2.1 RT-PCR的结果

*Survivin*基因RT-PCR扩增产物及阴性对照（PBS作为模板）分别取5 μL样本，经1.5%琼脂糖凝胶电泳分析，结果如图 1示：在250 bp-500 bp DNA marker（上海生工DL2000）之间显示的DNA条带与人Survivin基因的cDNA 429 bp相符合，阴性对照无条带出现。

**1:**
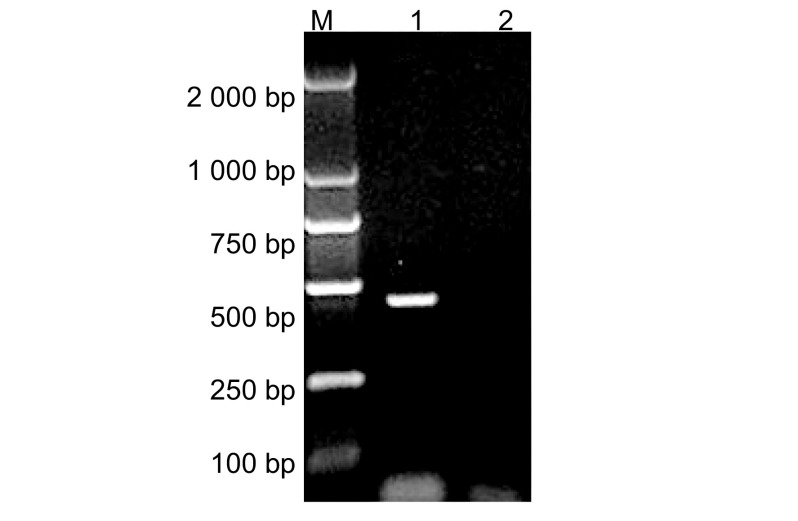
*Survivin*基因PCR扩增的结果。M：DNA marker；1：Survivin RT-PCR产物；2：阴性对照。 PCR amplification of Survivin. M: DNA marker; 1: RT-PCR product of Survivin; 2: Negtive control.

### 2.2 重组质粒的鉴定

重组质粒pGEMT-Teasy/Survivin双酶切后得到500 bp左右大小的片段，亚克隆至pET30a(+)中，*Nde*Ⅰ及*Nde*Ⅰ和*Xho*Ⅰ酶切pET30a(+)/Survivin重组质粒，10 μL酶切产物样本经1.5%琼脂糖凝胶电泳分析结果如图 2示，单酶切产物与线型pET30a(+)/Survivin质粒的位置大小一致，双酶切产物在400 bp-500 bp之间有一条明显片段。酶切产物测序结果经BLAST比对，与GenBank上所报道的人*Survivin*基因相一致，pET30a(+)/Survivin表达载体构建成功。

**2:**
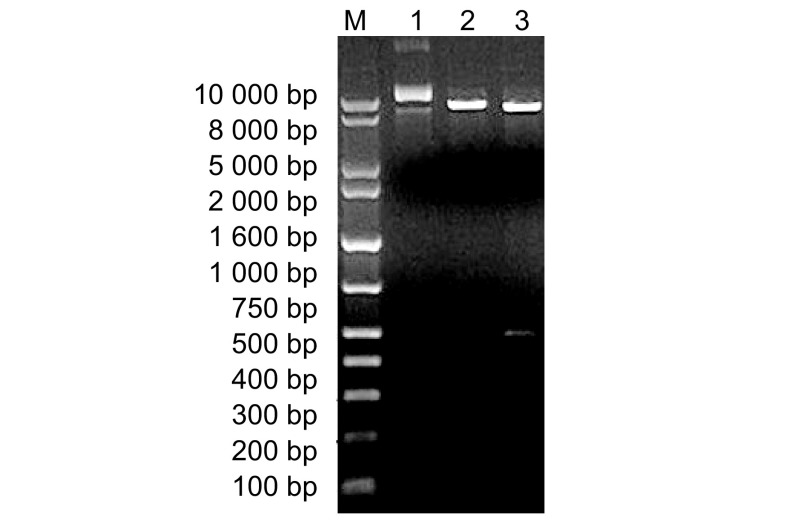
重组质粒的酶切鉴定。M：DNA marker；1：pET30a(+)/Survivin质粒；2：单酶切产物；3：双酶切产物。 Restriction enzyme digestion analysis of the recombinant plasmid. M: DNA Marker; 1: pET30a(+)/Survivin plasmid; 2: Single restriction enzyme digestion; 3: Double restriction enzyme digestion.

### 2.3 Survivin蛋白的表达及鉴定

菌液经不同浓度IPTG诱导，收集并制备样本，点样于12%SDS-PAGE电泳，分析结果如图 3示，在约19 kDa处出现一新蛋白条带，其含量约占菌体总蛋白的50%，而空载体转化的菌株无相应蛋白条带出现，说明新生蛋白条带非空载体pET30a(+)重组到*E.coil* BL21(DE3)所表达的蛋白；pET30a(+)/Survivin未诱导菌株同样无相应蛋白条带出现，说明在无IPTG诱导情况下，重组至*E.coil* BL21(DE3)中的Survivin基因是不能在*E.coil* BL21(DE3)中表达；而在IPTG诱导下，重组至*E.coil* BL21(DE3)中的*Survivin*基因可成功表达Survivin蛋白；由于诱导蛋白表达的IPTG浓度的不同，导致Survivin蛋白表达量不同，以0.4 mmol/L IPTG浓度诱导*E.coil* BL21(DE3)时，Survivin蛋白表达量最高，为最优蛋白表达条件。Western blot分析结果如图 4示：抗His标签抗体和抗Survivin单克隆抗体孵育的新生蛋白条带处，均可见与Survivin蛋白分子量大小相一致的特异性抗原、抗体反应条带，进一步证实新生条带为Survivin蛋白。

**3:**
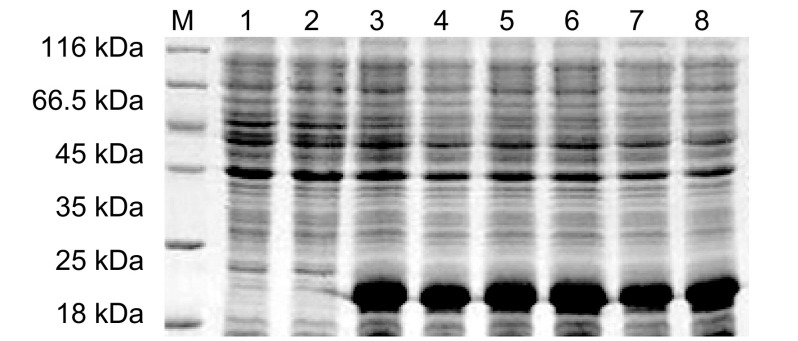
不同浓度IPTG诱导的Survivin的表达。M：Protein marker；1：空载体pET30a(+)；2：0 mmol/L IPTG；3：0.1 mmol/L IPTG；4：0.2 mmol/ L IPTG；5：0.3 mmol/L IPTG；6：0.4 mmol/L IPTG；7：0.8 mmol/L IPTG；8：1 mmol/L IPTG。 The expressions of pET30a(+) and pET30a(+)/Survivin induced at different IPTG concentraions. M: Protein marker; 1: pET30a(+); 2: 0 mmol/L IPTG; 3: 0.1 mmol/L IPTG; 4: 0.2 mmol/L IPTG; 5: 0.3 mmol/L IPTG; 6: 0.4 mmol/L IPTG; 7: 0.8 mmol/L IPTG; 8: 1 mmol/L IPTG.

**4:**
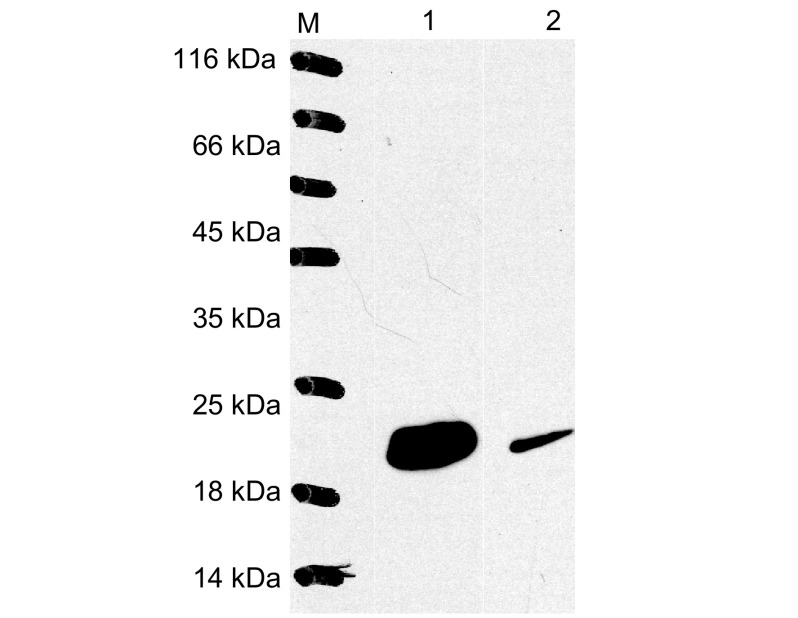
Survivin蛋白的Western blot的鉴定。M：Protein marker；1：抗his单克隆抗体孵育；2：抗survivin单克隆抗体孵育。 The Western blot result of Survivin. M: Protein marker; 1: Anti-his monoantibody; 2: Anti-Survivin monoantibody.

### 2.4 Survivin融合蛋白的纯化

利用细胞分层分级技术确定Survivin蛋白在*E.coil* BL21(DE3)中是以包涵体形式大量表达。Survivin融合蛋白的C端含有6×his标签，采用镍离子亲和层析方法纯化蛋白，利用不同浓度咪唑洗脱液洗脱蛋白，并将超声后的上清液及不同浓度的咪唑洗脱液收集行12% SDS-PAGE电泳如图 5示，菌体冰浴超声后上清中未见Survivin蛋白条带，说明Survivin蛋白未表达在细菌的周质腔中；而在含有20 mmol/L、30 mmol/L、50 mmol/L咪唑的洗涤液中可见大量的杂蛋白，说明低浓度咪唑起到了去除杂蛋白的作用；而当洗脱液中的咪唑浓度为100 mmol/L时，可将Survivin蛋白洗脱下来，经透析、浓缩获得纯度在95%以上Survivin蛋白。

**5:**
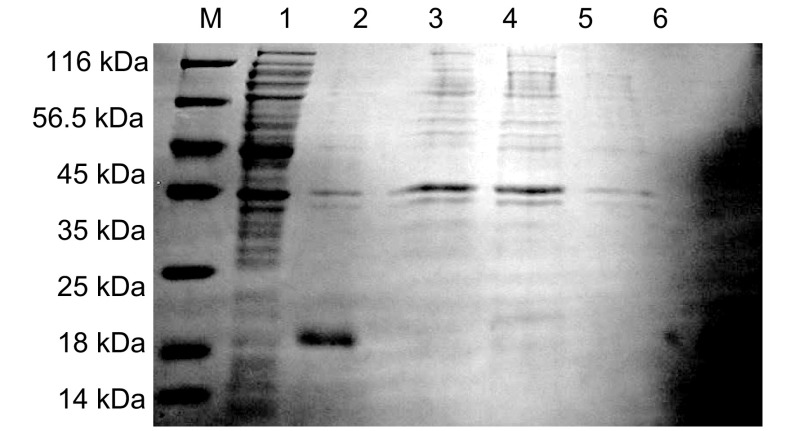
Survivin蛋白的纯化。M：Protein marker；1：超声后上清；2：溶解的包涵体；3：20 mmol/L咪唑洗脱液；4：30 mmol/L洗脱液；5：50 mmol/L咪唑洗脱液；6：纯化的Survivin蛋白。 Purification of Survivin protein. M: Protein marker; 1: Supersonical supernatant; 2: Solved inclusion; 3: 20 mmol/L iminazole eluention; 4: 30 mmol/L iminazole eluention; 5: 50 mmol/L iminazole eluention; 6: The purificationof Survivin protein.

### 2.5 NSCLC患者血清中Survivin自身抗体的检测及临床意义

利用间接ELISA方法检测血清中Survivin自身抗体的含量，其中89例健康志愿者的*A*_450_最高值为0.683，最低值为0.162，平均值为0.427，标准差为0.115。以Mean+2SD作为阳性血清样品的评判标准，*A*_450_≥0.657即可判为Survivin自身抗体阳性；反之，则判为阴性。Survivin在215例NSCLC患者血清的阳性率为19.5%，特异性为88.9%。而在20例肺部良性疾病患者血清中Survivin抗体检测均为阴性，由于良性疾病的标本例数太少，统计学分析结果不能充分代表肺部良性疾病患者情况，只能以此作为参考。

Survivin自身抗体与NSCLC患者病理参数之间是否存在统计学意义上的相关性，目前还存在着争议，本实验认为Survivin自身抗体在NSCLC患者血清中的含量与肺部原发肿瘤的大小存在着明显的统计学意义上的相关性（*P* < 0.05），即NSCLC患者血清中Survivin自身抗体检测为阳性时，肺部原发肿瘤体积≥3 cm的可能性越大。Survivin自身抗体在NSCLC患者中血清中的含量与肺部肿瘤存在远处转移也具有统计学意义上的相关性（*P* < 0.05），即NSCLC患者血清中Survivin自身抗体检测为阳性时，NSCLC患者的肿瘤易发生远处转移。而Survivin自身抗体在NSCLC患者中血清中的含量与NSCLC患者的年龄、性别、是否吸烟、病理类型、淋巴结转移以及临床分期均无相关性，具体数据见表 1。

**1:** 临床病理参数与Survivin自身抗体表达之间的关系 The relationship between clinicopathological parameters and the expression of anti-Survivin antibody in NSCLC

Clinical characteristics	Positive (*n*)	Negative (*n*)	Total	χ^2^	*P*
Age (yrs)				1.559	0.239
< 60	18	88	106		
≥60	26	83	109		
Gender				0.111	0.856
Male	31	116	147		
Female	13	55	68		
Smoking status				1.900	0.229
Smoking	30	97	127		
Non-smoking	14	74	88		
Pathological type				0	1.000
Squamous cell carcinoma	17	66	83		
Adenocarcinoma	27	105	132		
Primary tumor				13.60	< 0.001^*^
< 3 cm	10	91	101		
≥3 cm	34	80	114		
Lymphatic metastasis				1.792	0.235
No	24	74	98		
Yes	20	97	117		
Distant metastasis No	34	100	134	5.264	0.024^*^
Yes	10	71	81		
Stage				0.577	0.442
Ⅰ/Ⅱ	13	41	44		
Ⅲ/Ⅳ	31	130	161		
^*^: *P* < 0.05.					

### 2.6 肿瘤标志物的联合检测

本实验通过对215例NSCLC患者病历资料的整理及Survivin自身抗体在NSCLC患者血清中表达情况进行统计分析发现，CEA与Survivin自身抗体联合检测的阳性率为42.79%，明显高于癌胚抗原（carcinoembryonic antigen, CEA）与神经元特异性烯醇化酶（neuronspecific enolase, NSE）、鳞状细胞癌抗原（squamous cell carcinoma, SCC）、细胞角蛋白（cytokeratin, CYFRA）、胃泌素释放肽前体（pro-gastrin releasing peptide, ProGRP）的联合检测的阳性率，具体数据见表 2。

**2:** 几种肿瘤标志物在NSCLC患者中的联合检测的结果 The combined detection result of tumor markers in NSCLC

Combinationsoftumormarkers	Positivecases (*n*)	Positive rate (%)
CEA+Anti-Survivin	92	42.79
CEA+NSE	66	30.70
CEA+NSE+SCC	73	33.95
CEA+NSE+SCC+CYFRA	77	35.81
CEA+NSE+SCC+CYFRA+ProGRP	78	36.28

## 3 讨论

Survivin是凋亡抑制蛋白家族中的最强的凋亡抑制因子。人*Survivin*基因定位于17q25，全长1 447 kb，含有4个外显子和3个内含子，编码142个氨基酸，蛋白相对分子质量约16.5 kDa。Survivin蛋白几乎表达于所有恶性肿瘤，而在成人终末分化组织不表达或低表达。肿瘤相关的自身抗体同肿瘤相关抗原一样与肿瘤的发生、发展存在着密切联系。目前研究显示在许多种癌症中均可以检测到Survivin自身抗体的存在。为了探索Survivin自身抗体在NSCLC患者中可能存在临床诊断价值，本实验成功构建了原核表达载体pET30a(+)/Survivin，诱导表达、纯化纯度在95%以上的Survivin融合蛋白，并建立间接ELISA方法对215例NSCLC患者、89例健康对照以及20例肺部良性疾病患者进行Survivin自身抗体的血清学检测，结果显示Survivin自身抗体在NSCLC患者血清中检测的阳性率为19.5%，特异性为88.9%，与目前的研究结果相类似。Yagihashi等^[6]^利用间接ELISA方法检测31例NSCLC患者血清中Survivin自身抗体，其阳性率为58.1%；在Rohayem等^[7]^研究显示51例肺癌患者的血清中Survivin自身抗体的阳性率为21.6%；而Karanikas等^[8]^研究结果显示Survivin自身抗体在76例NSCLC患者血清中的阳性率为7.7%。几个实验组所得的Survivin自身抗体的阳性率均与本实验的Survivin自身抗体在肺癌患者血清中检测的阳性率不一致，分析其原因可能是由于样本来源、样本大小以及使用的评判标准不同，在实验结果上可能存在着一些差异，但在肺癌的研究中Survivin自身抗体仍是个比较有意义的肿瘤标志物。到目前为止，Survivin自身抗体在NSCLC患者血清中表达量至今仍未能得到一个公认的阳性定量标准，还需大量实验进一步研究。

Survivin自身抗体与癌症患者病理参数之间是否存在统计学意义上的相关性，目前还存在着争议，Karanikas等^[8]^研究结果显示，Survivin自身抗体与NSCLC患者的病理参数之间的相关性无统计学意义上，仅能作为一个预后及药效评价的参考指标。而本实验认为NSCLC患者血清中Survivin自身抗体检测为阳性时，肺部原发肿瘤体积≥3 cm的可能性越大，NSCLC患者的肿瘤更易发生远处转移。肿瘤的转移与原发肿瘤的大小的联系紧密，原发肿瘤体积越大，肿瘤易发生转移。但是本实验经相关分析得出原发肿瘤大小与远处转移未存在明显相关性（*r*=0.167, *P* > 0.05），因此本实验认为NSCLC患者血清中的Survivin自身抗体为阳性时，NSCLC患者的肿瘤更易发生远处转移。这一结论为今后Survivin自身抗体能否成为术前评估、肿瘤恶化程度及肿瘤远处转移的参考指标提供了潜在的研究价值。

20例肺部良性疾病患者血清中Survivin抗体检测均为阴性，由于肺部良性疾病的标本例数太少，不能充分说明Survivin自身抗体特异性较高这一优势，还需大量实验进一步证实。

Survivin自身抗体在215例NSCLC中的阳性检出率不是很高，为提高肿瘤的检测的敏感性，肿瘤标志物的联合检测成为肿瘤临床检测的趋势。目前以CEA、NSE、SCC、CYFRA、ProGRP的联合应用最为广泛，但是这种组合并非是针对NSCLC的临床检测所设计，敏感性不是很高。为了提高诊断的质量、寻找特异性和敏感性较高的肿瘤标志物，肿瘤标志物的不同组合的联合检测成为新的研究热点。本实验通过对215例NSCLC患者病历资料的整理及Survivin自身抗体在NSCLC患者血清中表达情况进行统计分析发现，CEA与Survivin自身抗体联合检测的阳性率为42.79%，明显高于CEA与NSE、SCC、CYFRA、ProGRP的联合检测的阳性率，大大提高了肿瘤标志物在NSCLC检测的敏感性，为Survivin自身抗体可能应用于肺癌的临床检测提供了广阔的应用前景。

总之，Survivin自身抗体作为NSCLC诊断的新亮点，其潜在价值越来越引起肿瘤研究者的广泛关注。Survivin自身抗体与其它肿瘤标志物的联合检测在肿瘤临床诊断上具有广阔的研究前景，尤其是对于高危人群的筛选和肿瘤的早期诊断上，Survivin自身抗体潜在的应用价值还需不断的探索。
